# Dementia and cognitive disorder identified at a forensic psychiatric examination - a study from Sweden

**DOI:** 10.1186/s12877-017-0614-1

**Published:** 2017-09-18

**Authors:** Anette Ekström, Marianne Kristiansson, Karin Sparring Björkstén

**Affiliations:** 1 Department of Neurobiology, Care Sciences and Society, Karolinska Institutet, Stockholm, Sweden; 2 Department of Geriatrics, Memory Section, Praktikertjänst N.Ä.R.A., Dalarövägen 6, Box 550, SE-136 25 Haninge, Sweden; 3 Department of Clinical Neuroscience, Centre for Psychiatry Research, Karolinska institutet, Stockholm, Sweden; 4 National Board of Forensic Medicine, BOX 4044, SE-141 04 Huddinge, Sweden; 5 Psychiatry South Stockholm, Ledning & Administration, Box 5040, SE-121 05 Johanneshov, Sweden

**Keywords:** Forensic psychiatric examination, Dementia, Cognitive disorder, Co-morbidity-alcohol-elderly

## Abstract

**Background:**

Few studies have addressed the relationship between dementia and crime. We conducted a study of persons who got a primary or secondary diagnosis of dementia or cognitive disorder in a forensic psychiatric examination.

**Methods:**

In Sweden, annually about 500 forensic psychiatric examinations are carried out. All cases from 2008 to 2010 with the diagnoses dementia or cognitive disorder were selected from the database of the Swedish National Board of Forensic Medicine. Out of 1471 cases, there were 54 cases of dementia or cognitive disorder. Case files were scrutinized and 17 cases of dementia and 4 cases of cognitive disorder likely to get a dementia diagnosis in a clinical setting were identified and further studied.

**Results:**

There were 18 men and 3 women; Median age 66 (*n* = 21; Range 35–77) years of age. Eleven men but no women had a previous criminal record. There were a total of 38 crimes, mostly violent, committed by the 21 persons. The crimes were of impulsive rather that pre-meditated character. According to the forensic psychiatric diagnoses, dementia was caused by cerebrovascular disorder (*n* = 4), alcohol or substance abuse (*n* = 3), cerebral haemorrhage and alcohol (*n* = 1), head trauma and alcohol (*n* = 2), Alzheimer’s disease (n = 2), Parkinson’s disease (n = 1), herpes encephalitis (n = 1) and unspecified (3). Out of four persons diagnosed with cognitive disorder, one also had delusional disorder and another one psychotic disorder and alcohol dependence. An alcohol-related diagnosis was established in ten cases. There were only two cases of Dementia of Alzheimer’s type, one of whom also had alcohol intoxication. None was diagnosed with a personality disorder. All but one had a history of somatic or psychiatric comorbidity like head traumas, stroke, other cardio-vascular disorders, epilepsy, depression, psychotic disorders and suicide attempts. In this very ill group, the suggested verdict was probation in one case and different forms of care in the remaining 20 cases instead of prison.

**Conclusions:**

Few cases of dementia or cognitive disorder were identified by forensic psychiatric examinations. All but one suffered from a variety of serious mental and medical conditions affecting the brain. Alcohol abuse was prevalent.

## Background

Understanding the impact of dementia has not been in focus in forensic psychiatry since criminal behaviour often starts at young age. Few studies have focussed on the mental condition of elderly criminals. A study from California showed that elderly criminals often were treated more harshly initially but more often got a milder verdict [1]. A Swedish study found that only <3% forensic psychiatric examinations concerned persons 60 years or older [2]. In 7% of those, dementia was found. In a register-based study from Finland, homicide offenders aged 60 or older had less drug dependence and personality disorders and more dementia and physical illnesses than younger homicide offenders [3]. In forensic psychiatric examinations in Turkey, most persons >60 years of age were diagnosed with schizophrenia or delusional disorder [4]. In South Carolina, violent crimes were common among persons >60 years of age that went through a forensic psychiatric examination [5]. In Korea, less than 1% of the patients in a forensic psychiatric hospital were diagnosed with dementia: Alzheimer patients being convicted for theft and patients with vascular or alcohol-related dementia for multiple violent crimes under influence of alcohol [6].

There are a few reports on persons with dementia committing murder [7, 8]. In murder-suicide cases by elderly couples, the victim often suffers from dementia and not the spouse committing the murder [9, 10].

Does dementia actually decrease the risk of committing crimes? Neurocognitive symptoms like memory impairment, reduced executive function, lack of judgement and language difficulties are hall-marks of dementia. Symptoms vary over time and with the type of dementia, comorbid diseases, medication, environment and care provided. Symptoms can separately or in combination contribute to criminal behaviour and actions.

Some persons with dementia become withdrawn and careful since they experience their shortcomings. Reduced executive function, that is common in Alzheimer’s disease, results in a passive behaviour, not likely to enhance planning and organizing a crime.

Focal prefrontal damage seems associated with an impulsive subtype of aggressive behaviour and persons with Frontal Lobe Dementia are at increased risk for violent crimes [11]. In a retrospective medical record review from a memory and ageing centre in California, patients with the behavioral variant frontotemporal dementia and semantic variant of primary progressive aphasia more often committed crimes than those with Alzheimer’s disease [12].

When a Swedish Court of Law finds a person guilty of a crime and the verdict is prison, it is possible to presentence a forensic psychiatric examination if the person has shown signs of mental disorder. The forensic psychiatric examination should establish whether the person suffered from a “severe mental disorder”, a judicial concept, at the time of the crime, and at the time of the examination. From a forensic point of view, a “severe mental disorder” is not related to diagnoses but to effects of disorders: 1) Psychotic effects or 2) Severe depression with suicidal behavior at the time of the crime or 3) Severe personality disorder with recurrent episodes of psychotic behavior and decreased psychosocial functioning or 4) Mental disorder with marked compulsiveness and decreased psychosocial functioning or 5) Severe dementia, severe mental retardation and severe brain damage. Mental disorders may be considered severe by the public and by the general health care system and but not from a forensic psychiatric point of view. If the person was in a state of severe psychiatric disorder at the time of the crime, the verdict can be forensic psychiatric care instead of prison. The court should also decide on whether cessation of forensic care be decided by the forensic psychiatrist or by a court procedure (“forensic care with separate discharge review”).

The National Board of Forensic Medicine is responsible for the examinations, not the health care system. About 500 persons annually in Sweden undergo a forensic psychiatric examination. A forensic psychiatric examination is performed by a team of forensic psychiatrists, psychologists, forensic social investigators and nursing staff.

If a crime is committed by a person with dementia or other severe mental disorder, the Swedish Law of Code of Judicial Procedure [13] allows the court to abstain from prosecution. There are, however, no statistics about how often this occurs. Probation, a non-custodial sanction that places the convicted person under supervision, is another possible verdict.

Since the number of elderly and persons with dementia increase, it is likely that there will be more crimes in this group.

### The aim of the work

The aim of this retrospective study was to characterize a group of persons with dementia undergoing forensic psychiatric examination to increase the understanding for risk factors for crime in persons with dementia.

## Methods

Cases were selected from the database of the Swedish National Board of Forensic Medicine including all Sweden. There were a total of 1471 forensic psychiatric examinations in 2008–2010. All cases from 2008 to 2010 with a primary or secondary diagnosis of dementia or cognitive disorder according to the DSM-IV classification [14] (290.0, 290.10–13, 290.20–21, 290.3, 290.40–43, 291.2, 292.82, 294.0–1, 294.8–10, 331.0–1, 631.0) were selected.

Since the criteria for dementia are inexact, we included cognitive disorder since we believed that there might be cases of dementia in this group. A total number of 54 cases were found, and the complete files were further scrutinized by two researchers with extensive clinical experience of dementia.

The first part of the scrutiny was to find the cases that would correspond to a dementia diagnosis in a clinical setting, and 27 cases were excluded. Out of the 27 cases excluded, 8 persons had 294.9 (cognitive disorder not otherwise specified) as primary diagnosis, and 18 persons had 294.9 (cognitive disorder not otherwise specified) as secondary diagnosis. In one case, the diagnosis was 294.8 (amnestic disorder not otherwise specified) as secondary diagnosis.

Five additional cases with DSM IV diagnosis 294.8–9 (amnestic disorder not otherwise specified) (cognitive disorder not otherwise specified) were excluded since the dementia work-up was incomplete from a clinical point of view or the individual was using drugs. In a clinical setting, those patients would be welcome for a dementia work-up after a period of no abuse. It thus remained a total of 22 cases. In one case, the same individual had undergone two forensic psychiatric examinations with the same result. One of them was therefore excluded yielding a total number of 21 cases.

The conclusions from the extensive psychological testing and the complete notes from physicians, nursing staff, social workers, and occupational therapists were scrutinized. Detailed data from 21 case files were extracted from the case files: Age, gender, year of birth, marital status, number of children, living conditions, education, profession, need of help with activities of daily life, smoking, use of alcohol and drugs at the time of the crime and otherwise, the current crime, previous crimes, all relevant previous and present medical data including pharmacological treatment and the results of a magnetic resonance tomography (MRI), computerized tomography (CT) or cerebral blood flow (CBF) of the brain, electroencephalogram (EEG), and laboratory tests, results from the neuropsychological tests and observation (impaired memory, impaired executive functions and judgement, impaired ability to understand instructions, impaired knowledge of time, hallucinations, delusions, delirium, aphasia, dyscalculia, prosopagnosia, agnosia, apraxia, Mini Mental State Examination score (MMSE) all diagnoses in the current and previous forensic psychiatry examination, former forensic psychiatric care, verdict and recommendation from National Board of Forensic Medicine. Due to the small size of the study, statistics were not needed.

## Results

### Demographic data

There were 21 subjects, 18 men and 3 women aged 35–77 (Median 66) years of age. Table 1 shows age, gender and living conditions at the time of the forensic psychiatric examination. To give an idea of the severity of dementia, deficiencies regarding orientation (*n* = 16) and primary ADL function (*n* = 15) observed at the forensic psychiatric examination are demonstrated in the table.

**Table 1:** Demographic data and severity of dementia in the 21 persons

Years of age	35	42	49	50	55	56	56	57	59	61	66	67	67	68	68	68	69	73	76	77	75
Gender	m	m	m	f	m	m	m	m	f	m	m	m	m	m	m	M	m	f	m	m	m
Living alone (A) or with spouse/partner (W)	A	W	A	W	A	W	A	W	A	A	A	W	A	A	A	W	W	A	A	W	W
Type of living*	HL	–	NH	–	NH	HL	HL	–	ALF	–	NH	–	–	–	C	–	–	HL	–	–	–
Dementia diagnosis already known	–	–	–	–	yes	–	–	–	–	–	yes	–	–	–	–	–	–	yes	–	–	–
Community services at home	yes	yes	yes	–	yes	–	yes	–	yes	–	yes	?	–	yes	–	–	–	yes	–	yes	?
Orientation deficiencies observed	yes	yes	yes	yes	yes	yes	–	–	yes	yes	yes	–	yes	yes	?	yes	yes	–	yes	yes	yes
Primary ADL deficiencies observed	yes	yes	yes	yes	yes	yes	–	–	–	yes	yes	yes	yes	yes	–	–	yes	yes	yes	?	yes

*Private home (−), Nursing Home (*NH*), Assisted Living (*AL*), Homeless (*HL*), Caravan (*C*)

Table 1 shows age, gender, whether living alone or in a relationship, type of living and community services at home. The three subjects previously diagnosed with dementia are shown in the table. To give an idea of the level of dementia, deficiencies regarding orientation and primary ADL function observed during the forensic psychiatric examination.

All persons had basic school education: elementary school (*n* = 9), secondary school (*n* = 4), vocational school (n = 4) and college level (n = 4). Seventeen persons were of Swedish origin, and four were immigrants. Fifteen persons had one or more children. A number of professions were represented; Air pilot, director, computer expert, film producer, secretary, tailor, carpenter, protected work, chef, kitchener, clerk, excavator operator, blue collar (*n* = 2), truck drivers (*n* = 3) and handymen (*n* = 4). One person was still working and another one was unemployed. The remaining persons either had a disability pension (*n* = 8) or old age pension (*n* = 11). Three persons had support according the “Swedish Act concerning Support and Service for Persons with Certain Functional Impairments”, which grants extensive supports for persons included. Nine persons had assistance with their personal finances; trustee (*n* = 6), administrator (*n* = 1) and good friend/relative (*n* = 2).

Nine persons had a driver’s license and in three cases, all men, the driver’s license had been withdrawn. One man had a license for weapons, and for another man, the license for weapon had been withdrawn.

### Diagnoses in forensic psychiatric examination

There were a total of 17 persons diagnosed with dementia, in 15 cases as primary diagnosis and in 2 cases as secondary diagnosis. Cognitive disorder not otherwise specified was the primary diagnosis in two cases and the secondary diagnosis in two cases. In ten cases, there was an alcohol-related diagnosis. None was diagnosed with a DSM-IV axis II personality disorder. The diagnoses established by the forensic psychiatric examination are demonstrated in Table 2.

**Table 2:** The diagnoses established by the forensic psychiatric examination

Primary diagnosis	n	Secondary diagnosis
Dementia not otherwise specified	3	Maladaptive stress reaction (1) Psychotic disorder not otherwise specified (1)
Cognitive disorder not otherwise specified	2	Alcohol dependence (1)
Dementia of the Alzheimer’s type	2	Alcohol intoxication (1)
Dementia due to head trauma and alcohol	2	Alcohol dependence (2)
Alcohol-induced persisting dementia	1	Alcohol dependence
Substance-induced persisting dementia	1	Alcohol dependence
Dementia due to Parkinson’s disease	1	
Dementia due to encephalitis by herpes virus	1	Alcohol dependence
Vascular dementia	1	
Vascular dementia with depressed mood	1	Post-traumatic stress disorder
Vascular dementia with delirium	1	
Multi-infarct dementia with delusions	1	
Alcohol dependence	1	Alcohol-induced persisting dementia
Psychotic disorder due to brain damage caused by alcohol	1	Cognitive disorder not otherwise specified and alcohol dependence
Delusional disorder	1	Cognitive disorder not otherwise specified
Personality change due to cerebral haemorrhage	1	Dementia due to cerebral haemorrhage and alcohol dependence

Table 2 shows the psychiatric diagnoses established by the forensic psychiatric examination in the 21 cases

### Previous crimes

Eleven men were found in the criminal record at the time of the forensic psychiatric examination. None of the three women were found in the criminal record. There were a total of 42 crimes. Seven men had committed violent crimes and four other men had committed non-violent crimes. The crimes were assault and battery (*n* = 7), unlawful threat (*n* = 6), violence and threat to public servant (*n* = 5), drunk driving (*n* = 4), unlawful driving/traffic offence (n = 4), violent resistance (*n* = 3), arson (*n* = 2), breach of domiciliary peace (n = 2), petty theft (n = 2), violation against the law of knifes (n = 2), alcohol-related crime (*n* = 1), crimes against the Narcotic Drugs Law (n = 1), preparation for assault (*n* = 1), molestation/abuse (n = 1), drunkenness at sea (n = 1). The verdicts were prison (*n* = 7), fines (*n* = 6), probation (*n* = 5), conditional sentence (*n* = 3), abstention from prosecution (*n* = 2), electronic tag (transmitter) (n = 1). Ten men had been convicted in a court of law and seven persons had been to prison one or several times. One man had previously been examined with a forensic psychiatric examination.

### Present crimes

There were a total of 38 crimes committed by the 21 persons. The crimes were unlawful threat (*n* = 6), aggravated assault (*n* = 5), aggravated rape against child/rape against child (*n* = 2), attempted arson/arson (*n* = 3), violence to public servant (n = 3), attempted murder (n = 2), petty theft/serious theft (n = 2), molestation (n = 2), serious violation of a woman’s integrity (n = 2), breach of domiciliary peace (n = 2), aggravated rape (*n* = 1), aggravated sexual assault against child (*n* = 1), attempted homicide (n = 1), serious violation of integrity (n = 1), violation of restraining order (n = 1), criminal mischief (n = 1), threat to public servant (*n* = 1), violent resistance (*n* = 1), carelessness endangering the public (n = 1). Victims of the crimes were partner (*n* = 10), public servant (*n* = 6), neighbour/acquaintance (*n* = 3), children of their own (2), grandchild (*n* = 1), unknown adult (*n* = 2), unknown child (n = 1). Twelve persons were under the influence of alcohol at the time of the crime. One woman had under influenced by alcohol assaulted and attempted to murder her partner (n = 1), and two women committed arson in their own homes (n = 2). Two of the women had earlier or ongoing alcohol abuse.

### Psychiatric history and co-morbidity

Somatic or psychiatric comorbidity were found in all cases except one. In this group of 21 persons, 16 persons had ongoing treatment with prescription drugs at the time of the forensic psychiatric examination.

The psychiatric diagnoses were depression (*n* = 6), delirium tremens (*n* = 4), other psychotic symptoms (n = 4), delusional disorder (*n* = 1), post-traumatic stress disorder due to torture and (n = 1) and panic disorder (n = 1). Three men and one woman had a history of one or more suicide attempts (hanging, jumping from heights, drowning, intake of caustic soda, poisoning). When admitted to forensic psychiatric examination, 13 persons had a history of previous or ongoing alcohol abuse and three persons had earlier or ongoing abuse of illegal drugs. Several persons used psychiatric prescription drugs; Sleeping pills (*n* = 7), serotonin re-uptake-inhibitors (*n* = 6), antipsychotic drugs (*n* = 5), tri-cyclic antidepressants (*n* = 1), sedative drugs (n = 1), and acamprosate (n = 1).

### Medical history

All but one had one or several previous or ongoing medical conditions affecting the brain negatively: Epilepsy (*n* = 7), previous stroke (*n* = 6), hearing impairment (n = 6), head trauma with concussion (n = 6), basilar skull fracture (*n* = 3), traumatic bleeding of the brain (n = 3), previous CNS infections (*n* = 2), cirrhosis of the liver (n = 2), leukoencephalopathy (*n* = 1), Parkinson’s disease (*n* = 1), fibro-myalgia (n = 1). There were also a number of cardiovascular and metabolic disorders; Hypertension (*n* = 6), diabetes (*n* = 4), heart failure (*n* = 3), carotid stenosis (n = 1), cardiac arrhythmia with pace-maker (n = 1), hyperlipidaemia (n = 1). In addition, there was one case of prostatic cancer and a number of other common medical problems.

Five persons were taking anti-epileptic drugs and five were taking anti-hypertensive drugs. Two persons used warfarin and three either statins, nitro-glycerine or insulin. In addition, seven persons were taking hormone or vitamin supplements and two proton pump inhibitors.

### Previous dementia assessment

Seven persons had previously gone through a dementia assessment and three of them had been diagnosed with dementia already before the forensic psychiatric examination. The diagnoses were alcohol-induced persisting dementia (*n* = 2) and dementia not otherwise specified (*n* = 1). An additional person had started a dementia assessment before the forensic psychiatric examination took place.

### Symptoms of dementia in the running text of the case files

We scrutinized the running text of the files for mentioning a number of common symptoms of dementia. Not surprisingly, “Lack of judgment” was specifically mentioned in all the 21 cases and “No insight in illness” in 16 cases.

Major cognitive symptoms appeared in all the cases: “Impaired memory” (*n* = 20), “Impairment of executive function” (*n* = 19), “Impaired ability to understand instructions” (n = 19), “Impaired knowledge of time” (*n* = 17), “Inability to reason” (*n* = 15), “Problems to handle ADL” (n = 15), “Disorientation” (*n* = 12), “Aphasia” (*n* = 10), “Prosopagnosia” (*n* = 8) and “Dyscalculia” (*n* = 7).

“Agitated/aggressive behaviour” was mentioned in 16 cases and overtly psychotic symptoms like “Theories of conspiracy” in 11 cases, “Delusions” in seven cases and “Hallucinations” in two cases. “Delirium at some time” was mentioned in 19 cases, and “confabulation” in nine cases. Fourteen persons were “Isolating themselves” and four showed “Symptom of depression”. “Affect incontinence” was mentioned in four cases.

### Examinations performed

All persons were examined by a physician and a psychologist to a lesser or greater extent. Sixteen of the 21 persons were examined with brain computerized tomography scan or/and magnetic resonance imaging, fourteen of whom with pathological results. Neuroimaging revealed a variety of focal brain lesions after stroke, trauma, and surgery as well as focal and generalized brain atrophy. In addition, there was one case of widespread white matter lesions and another case of sequela after herpes encephalitis. In the latter case, EEG showed gross abnormalities and epileptic activity, and in another person who had suffered a stroke, reduced cortical function in the right temporal region was found. Table 3 summarizes the different examinations.

**Table 3:** The different examinations performed as part of the 21 forensic psychiatric examinations

Examination	Performed	Pathologic Result
Assessment by a psychologist	21	21
Neurological examination	17	10
Computerized Tomography scan	15	10
Blood samples	12	4
Mini Mental State Examination	10	8
Blood pressure	9	5
Magnetic Resonance Imaging	7	6
Electro-Encefalogram (EEG)	7	2
Cerebral Blood Flow (CBF)	6	4
Clock test	1	1

Table 3 shows the different examinations performed as part of the 21 forensic psychiatric examinations and the number of pathological results

### Recommended verdict from National Board of forensic medicine

Forensic psychiatric care with separate discharge review (*n* = 10), forensic psychiatric care without separate discharge review (*n* = 6), care in a nursing home for persons with dementia (*n* = 2), Forensic psychiatric care at a nursing home (n = 1), ordinary care at a nursing home (n = 1), probation (n = 1).

## Discussion

The most striking finding of this study of forensic psychiatric diagnoses was that there were very few persons being diagnosed with dementia. This is in agreement with studies from countries like Finland, Korea, the U.S. and Turkey [3–6].

Although dementia primarily occurs at old age, half of the persons were younger than 65 years. Dementia of Alzheimer’s type constitutes approximately two thirds of all cases of dementia, but this was not reflected in the group of forensic psychiatric examination. In our study, only 2 persons (10%) had Dementia of Alzheimer’s type, one of whom also had alcohol intoxication at the time of the crime.

Alcohol is a well-known risk factor for violent crimes world-wide. Although we excluded those with alcohol abuse at the time of the forensic psychiatric examination, 48% of the cases had alcohol-related diagnoses. The ageing brain and especially a brain with a disease or previous trauma are also more sensitive to alcohol.

It is not surprising that many of the persons had a history of mental disorders, but all but one also had a history of serious medical disorders and/or traumas. Six persons (29%) had a history of head trauma with concussion and three additional persons (14%) had experienced traumatic brain haemorrhage. Six persons (29%) had a history of stroke and additionally five persons (24%) other evidence for cardiovascular disorder. Two persons (5%) had sequel after meningitis or encephalitis and seven persons (33%) had epilepsy. Three persons (14%) had a previously established diagnosis of dementia and 38% had previously gone through a clinical dementia work-up. The above-mentioned conditions are also risk factors for dementia. In spite of serious medical conditions, few patients were taking medication regularly.

There was no case of personality disorder in this study, perhaps due to small study size. Personality disorder may, however, in accordance with a Finnish study of psychopathy be less prevalent in elderly criminals compared to younger ones [3]. It is also possible that other diseases including dementia overshadowed a personality disorder.

Prefrontal damage seems to predispose for impulsive, aggressive behaviour and persons with frontal lobe dementia are at increased risk for violent crimes [11, 12]. There were, however, no cases of frontal lobe dementia in this material, perhaps due to the small numbers.

Our findings underline the importance of protecting the brain from trauma, disease, and alcohol as far as possible, which concerns every practicing physician. Medical help for alcohol abuse should be available also for elderly and persons with dementia. Many geriatricians and old age psychiatrists would appreciate collaboration with colleagues in forensic psychiatry when patients with dementia become dangerous. With the growing elderly population, forensic psychiatry could benefit from collaboration with those who work with dementia on a daily basis, and more knowledge about dementia and cognitive disorders should be included in the curriculum for forensic psychiatrists in training.

## Conclusion

In conclusion, we found that forensic psychiatric examinations seldom lead to a dementia diagnosis, and when it does, the majority of persons have a number of medical conditions affecting the brain. Alcohol is a strong risk factor for committing crimes in persons with or without dementia.

The crimes are mostly impulsive-aggressive as opposed to premeditated. A person with dementia may easily misunderstand a situation and act improperly. We believe that in many cases where persons with dementia are involved in crimes, it is apparent for anyone that they do not fully understand. It is likely that many cases are never taken to court, although no statistics is available on waiver of prosecution. In this very ill group, the suggested verdict was probation in one case and different forms of care in the remaining 20 cases instead of prison. Even without having committed crimes, those persons were in need of a lot of attention and care.
